# Sex differences in the associations between right heart structure and peak exercise capacity parameters in amateur cyclists

**DOI:** 10.3389/fphys.2024.1427101

**Published:** 2024-07-29

**Authors:** Michał Jakub Pytka, Remigiusz Andrzej Domin, Mikołaj Stanisław Żołyński, Jan Niziński, Tomasz Krauze, Andrzej Wykrętowicz, Przemysław Guzik

**Affiliations:** ^1^ Department of Cardiology–Intensive Therapy, Poznan University of Medical Sciences, Poznań, Poland; ^2^ University Centre for Sports and Medical Studies, Poznan University of Medical Sciences, Poznań, Poland; ^3^ Doctoral School, Poznan University of Medical Sciences, Poznan, Poland; ^4^ Department of Endocrinology, Metabolism and Internal Medicine, Poznan University of Medical Sciences, Poznań, Poland

**Keywords:** athlete’s heart, endurance exercise, echocardiography, oxygen consumption, right ventricle, right atrium, sex differences

## Abstract

**Introduction:** Right heart changes and their association with exercise capacity, including sex differences, are still being investigated. We analysed right heart structure and its relationship with exercise capacity parameters in amateur cyclists.

**Materials and methods:** A cross-sectional study involving 215 consecutive adult amateur cyclists, who underwent resting transthoracic echocardiography and a cardiopulmonary exercise test (CPET) to exhaustion was performed.

**Results:** The median age of participants was 29 years (IQR 24–37), 71% of them were men. The mean training time was 6 h/week, and 90% participated in vigorous or moderate physical activity. Men had larger right ventricular diameters (basal - RVD1, mid-cavity - RVD2 and longitudinal - RVD3) compared to women (40.9 vs. 37.6 mm, *p* = 0.0005, 28.7 vs. 26.3 mm, *p* = 0.03, 92.2 vs. 81.9 mm, *p* < 0.0001). Indexing for body surface area revealed comparable right atrial volume (RAVI) between sexes (24.1 vs. 22.7 mL/m^2^). Men achieved higher peak exercise capacity parameters [O_2_ pulse, oxygen consumption (VO_2_) and workload] in CPET. Multivariate linear regression models revealed a positive association between peak VO_2_, workload and O_2_ pulse with RAVI in women but not with RVD1 or RVD3. Conversely, these parameters showed a positive association with RVD3 and RVD1 but not with RAVI in men.

**Conclusion:** Sex differences exist in the relationship between right heart structural parameters and peak exercise capacity descriptors in amateur cyclists. Better exercise capacity during CPET to exhaustion is associated with greater RAVI in women but a greater RVD1 and RVD3 in men. These findings suggest different mechanisms of right heart adaptation to training in men and women.

## Graphical Abstract

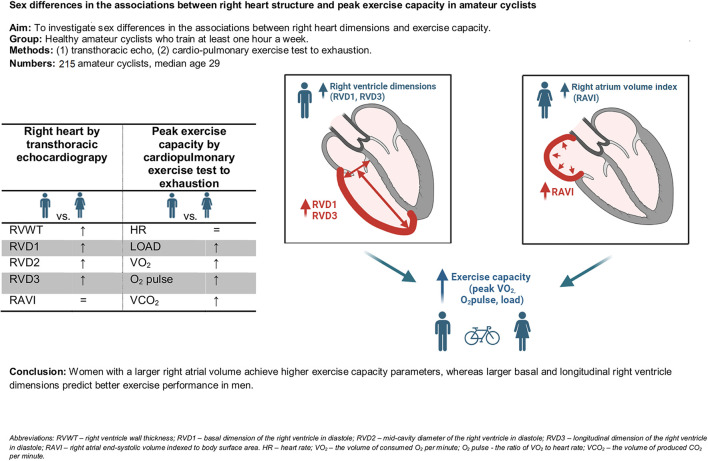

## 1 Introduction

The athletes’ heart develops in response to long-term training. Exercise and training type, volume, intensity, start age, duration of training hours, years of exercise and sports discipline influence the pattern of cardiac adaptation (Morganroth et al., 1975; Hellsten and Nyberg, 2015; Haykowsky et al., 2018; Bernardino et al., 2020; Conti et al., 2021; La Gerche et al., 2022; Qiu et al., 2022). This adaptation is additionally modified by the level of hemodynamic stress and duration of exposure (Haykowsky et al., 2018).

The heart adapts to exercise differently, depending on the type and intensity of physical activity (Hellsten and Nyberg, 2015; Bernardino et al., 2020; Conti et al., 2021; Qiu et al., 2022). Prolonged and repeated endurance exercises like cycling, increase blood flow through the heart and circulation. During endurance exercise, the right and left hearts pump approximately the same amount of blood, mainly resulting in eccentric ventricle dilation. Some concentric adaptation may also occur, making the ventricular walls thicker and stronger (Lewicka-Potocka et al., 2019; Churchill et al., 2021; Marsh et al., 2021).

Men and women differ in body shape, weight, size and composition. Men are often taller, heavier and have larger internal organs, including the heart (Bassett et al., 2020). They generally present better physical performance, endurance, greater muscle mass and strength and may be able to achieve greater absolute speed or power than women, both non-athletes and athletes (Bassett et al., 2020).

Studies of the athlete’s heart have mostly recruited men. Cardiac adaptations to exercise in women are sparsely explored. Both sexes exhibit cardiac adaptations to exercise, though differences between men and women are occasionally reported (D’Ascenzi et al., 2017; 2024; Colombo and Finocchiaro, 2018). Female athletes have more frequent left ventricle (LV) eccentric remodeling suggesting that cardiac adaptation to exercise is characterized by an increased chamber size rather than wall thickness (Colombo and Finocchiaro, 2018). However, their right heart is smaller, with thinner walls and lower LV mass than male athletes (D’Ascenzi et al., 2017).

In athletes, the right heart has been less studied than the left. On average, endurance athletes have increased right ventricle (RV) mass and volume compared to non-athletes or resistance athletes, which is caused by the greater hemodynamic stress in the RV during endurance exercise (La Gerche et al., 2011; Gargani et al., 2023). Structural parameters of the RV, such as basal dimension - RVD1, mid-cavity dimension - RVD2, and longitudinal dimension - RVD3 manifest greater changes than functional parameters in endurance athletes (Conti et al., 2021).

Exercise also causes structural changes in the right atrium (RA). However, this area lacks detailed research. (Conti et al., 2021). D'Ascenzi et al. reported normative reference values of the right heart in athletes (D’Ascenzi et al., 2017). Male athletes had higher upper limits for RV and RA dimensions than sedentary men. No conclusive data for women was shown, due to fewer available studies (D’Ascenzi et al., 2017). Directly comparing the right heart between male and female athletes is uncommon.

Endurance training, characterized by many hours of exercise, induces eccentric adaptation throughout the heart, including the RV and RA. Consequently, a larger RV and RA in athletes might indicate their higher exercise capacity. The existence of sex differences in RV and RA size, as well as the association between the right heart sizes and exercise capacity in endurance athletes, remains unclear (Conti et al., 2021; Ramcharan et al., 2023).

This study analyzed the right heart structure in healthy adult amateur cyclists according to sex and their relationship with peak exercise capacity parameters.

## 2 Materials and methods

### 2.1 Bioethical issues and data collection

The project was approved by the Bioethics Committee of the Poznan University of Medical Sciences (decision 693/20) and conducted according to the Declaration of Helsinki (Sawicka-Gutaj et al., 2022). All data were treated confidentially and anonymized for storage and analysis. The data were collected, stored, and analyzed in the Redcap data capture environment at Poznan University of Medical Sciences (https://redcap.ump.edu.pl).

### 2.2 Study group recruitment and participant consent

For this cross-sectional, observational study, we recruited 215 physically active healthy adults through a call issued for amateur cyclists interested in participating in a study to evaluate their cardiovascular function using ultrasound and exercise testing. Enrolment included consecutive volunteers who responded to the recruitment call and took place between December 2020 and April 2023. The minimum inclusion criteria were cycling for at least 1 hour per week to ensure a diverse sample of amateur athletes with varying levels of exercise capacity. Individuals with known chronic diseases or taking medications were excluded. Dietary supplements and oral contraceptives were allowed.

All participants were fully informed about the study, including the fact that their participation was voluntary and the option to withdraw at any time. Written informed consent was collected from each participant.

### 2.3 Health status

Health status was assessed based on medical history including chronic and acute illnesses and a family history of cardiovascular disease by physicians. The physical examination involved checking for signs of disease including auscultation of the lungs and heart and blood pressure. Electrocardiography was performed to identify abnormal heart rhythms, conduction or electrical disease, or signs of other cardiac abnormalities such as left ventricular hypertrophy. Resting transthoracic echocardiography assessed cardiac structure and function and excluded clinically relevant anomalies such as left ventricular hypertrophy, chamber dilatation, moderate to severe valvular stenosis or regurgitations, or contractile abnormalities. Six individuals were excluded due to such anomalies.

### 2.4 Anthropometric measurements and physical activity assessment

Anthropometric parameters, including height, weight, and body composition, were obtained using a stadiometer and a body composition analyzer (total four-frequency body impedance analyzer, *Tanita MC 180 MA, Tanita, Tokyo, Japan*). Participants completed the International Physical Activity Questionnaire (IPAQ) in order to assess physical activity category (Hagströmer et al., 2006) and additional questions regarding the number of cycling training sessions per week (training days per week) and their duration (hours of training per week). The estimated total MET-minutes (metabolic equivalent of task - minutes) were calculated according to IPAQ interpretation guidelines (Hagströmer et al., 2006) (MET-minutes are equivalent to kilocalories for a 60-kg person) and represented the total exercise volume of the participant.

### 2.5 Spirometry

Baseline spirometry was performed (*Vyntus CPX, Vyaire Medical, IL,* United States) in a sitting position after resting for 5 min. Forced expiratory volume in one second (FEV1) was measured and used to estimate the maximal voluntary ventilation (MVV) according to the formula FEV1*40 (Pritchard et al., 2021). MVV defined the limit of breathing reserve (BR) for the cardiopulmonary exercise test (CPET). Spirometry was performed with at least five repetitions to obtain results of adequate quality and the mean of the three best results was used for further analysis.

### 2.6 CPET

CPET was performed on a cycle ergometer (*Excalibur Sport 2, Lode, Groningen, Netherlands*) using a CPET system (*Vyntus CPX, Vyaire Medical, IL,* United States). The test was performed in a sports laboratory with a surface area of a 54 m^2^ and a height of 3.3 m. A central air conditioning system is set to keep stable conditions and maintain an average temperature of 20–21°C and a humidity level between 45% and 55%. Before each test, the CPET system was calibrated according to the manufacturer’s instructions. The tidal volume (TV) and the changes in the content of oxygen (O_2_) and carbon dioxide (CO_2_) in the respiratory air were measured continuously using a breath-by-breath method. Original measurements were recorded using a breath-by-breath approach and then averaged over eight preceding breaths. This method aligns with the manufacturer’s (Vyaire) recommendations and is commonly employed in CPET studies. Heart rate (HR) was recorded using a chest-strap heart rate monitor (*Polar H10, Polar, Kempele, Finland*), and data were transmitted by Bluetooth. HR measured by a heart rate monitor was automatically used to compute O_2_ pulse as the ratio of VO_2_ by HR. The averaged CPET values from the last 15 s of the rest and warm-up phases and the 15 s preceding the following time points: first (VT1) and second ventilatory thresholds (VT2), and peak exercise were used to avoid single result deviation. Peak HR, VO_2_, O_2_ pulse and load were used as indices of the peak endurance exercise performance.

Each participant underwent CPET to exhaustion according to the Association for Respiratory Technology & Physiology (ARTP) guidelines (Pritchard et al., 2021) using an individualized incremental ramp protocol. The protocol for each participant was tailored based on current physical performance which included regularity and intensity of exercise and estimated maximal workload. To customize the ramp protocol for each individual amateur cyclist, we leveraged the International Physical Activity Questionnaire (IPAQ) to estimate their fitness level. Based on the IPAQ category, different target power outputs were set for the maximal load after 10 min:

• Cyclists in the lower physical activity category aim for a maximal load of 2.5–3.5 W/kg.
• Cyclists in the moderate physical activity category target 3.5–4.5 W/kg.
• Cyclists in the high physical activity category aim for 4.5–5.5 W/kg.

This approach ensures an appropriate challenge for all cyclists, preventing the CPET from being too easy or demanding. It allows them to reach their maximum effort within 8–12 min. By tailoring the protocol this way, we account for individual fitness levels, guaranteeing that each cyclist is tested appropriately according to their capabilities.

The resting phase lasted 2 min, the adaptation phase (low-load pedaling) lasted 2 min and the warm-up phase lasted 3 min. The main phase of the test, the progressive exercise, aimed to optimally last 8–12 min (Pritchard et al., 2021). After maximal exercise the participant was further monitored for at least 10 min.

The incremental exercise phase, guided by the ramp protocol, was initiated and continued until the exhaustion. All subjects were actively encouraged to cycle to their maximum effort. The current cadence and load in Watts were displayed in front of the participants and they were allowed to manage the increasing load by increasing cadence, muscle strain, or both. The participants received a suggestion to start pedaling with a cadence of 60 rpm and gradually increase the cadence, but they were free to alter the cadence at their will. During the recovery phase, the participants stopped pedaling and rested seated.

An explanation of CPET parameters, which are used in our analysis is presented in the Supplementary Table S1.

VT1 and VT2 were determined by at least two physicians experienced in CPET analysis. For VT1, we used the V-slope method from the VO_2_ vs. VCO_2_ (the volume of produced CO_2_ per minute) relationship, VE/VO_2_ (ventilatory equivalent for O_2_) vs. load, and PetO_2_ (the end-tidal O_2_ tension in exhaled air) vs. load. For VT2, we used PetCO_2_ (the end-tidal CO_2_ tension in exhaled air) vs. load, VE/VCO_2_ V-slope from the VE vs. VCO_2_ plot, and VE/VCO_2_ (ventilatory equivalent for CO_2_).

### 2.7 Echocardiography (ECHO)

Resting transthoracic echocardiography (TTE) was performed according to the guidelines of the American Society of Echocardiography (Mitchell et al., 2019). A 3.5-MHz transducer was used on a Vivid E95 or E9 echocardiography machine from General Electric Healthcare Technologies (*Chicago, IL,* United States). In addition to the standard TTE measurements, a detailed analysis of the right heart was performed (D’Ascenzi et al., 2017; Jones et al., 2019) from the RV-focused 4-chamber view for right atrial volume (and calculating the right atrial end-systolic volume indexed to body surface area or RAVI–measured from a single plane 4-chamber view), right ventricular area in systole and diastole, right ventricular dimensions in diastole (RVD1, RVD2, RVD3).

**FIGURE 1:**
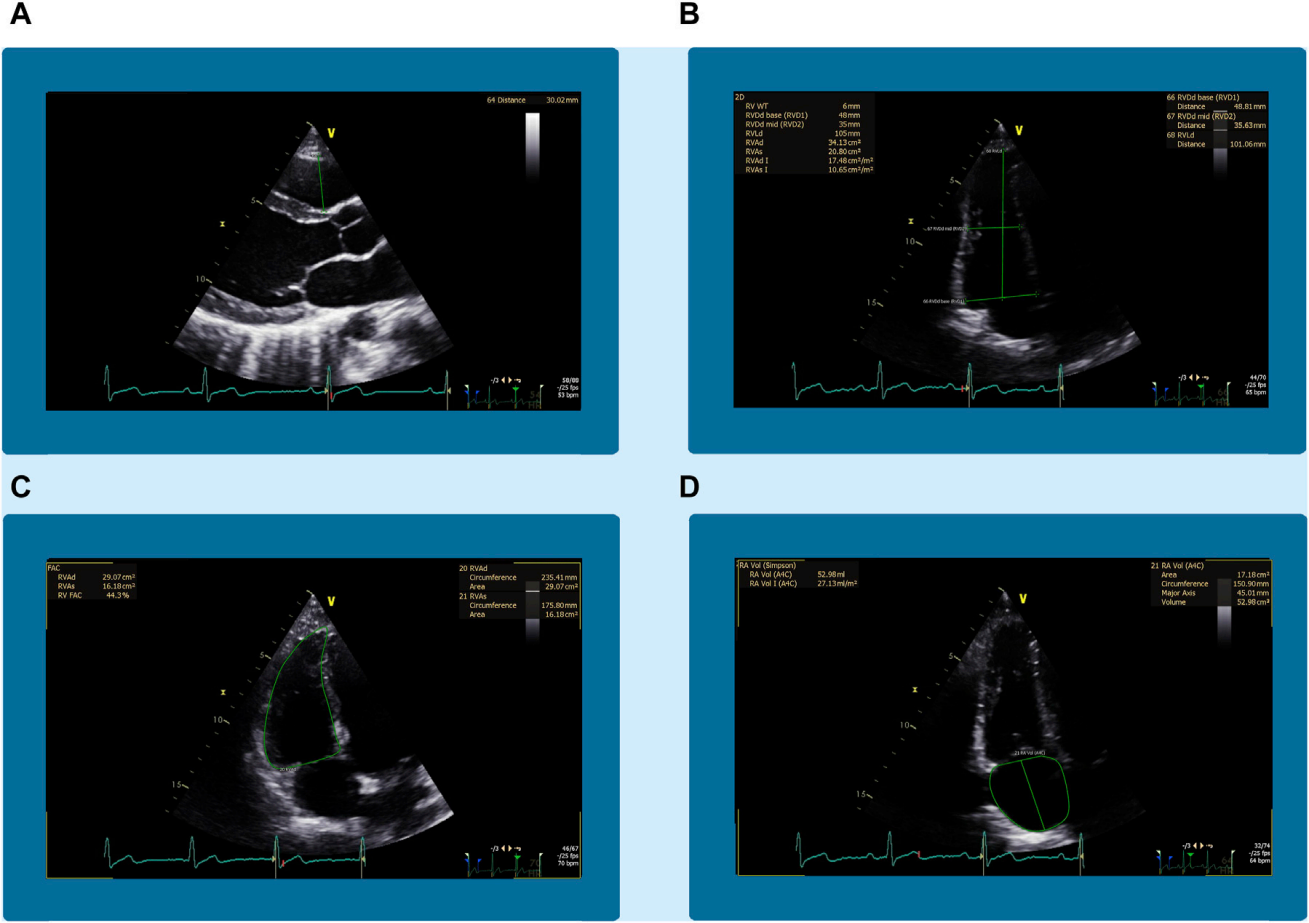
An example of the echocardiographic measurements from the right heart. Panel **(A)** shows the right ventricular outflow tract (RVOT) measured in the parasternal long-axis view. Panel **(B)** shows the basal (RVD1), mid-cavity (RVD2), and longitudinal (RVD3) diameters of the right ventricle (RV) in the four-chamber view of the right heart. Panel **(C)** shows the same view of the right ventricular end-diastolic area (RVEDA). Panel **(D)** shows the right atrial volume (RAV) in the four-chamber view focused on the right heart.

The right ventricular wall thickness was measured in the subcostal view during breath-hold. The images and cine loops were exported to TOMTEC Imaging Systems (*TOMTEC-ARENA Build No. 544347, TOMTEC Imaging Systems GmbH, Unterschleissheim, Germany, distributed by Phillips, Amsterdam, Netherlands*) for post-processing analysis and measurements. An example of ECHO measurements is shown in Figure 1.

### 2.8 Statistical analysis

The normality of the data distribution was tested using Q-Q plots and the D'Agostino-Pearson test (Guzik and Więckowska, 2023). Normally distributed data were reported as mean ± standard deviation (SD), and non-normally distributed data as median and 25th and 75th percentiles (IQR - interquartile range). Comparisons between men and women for unpaired data were made using either the Student’s t-test or the Mann-Whitney test depending on the data distribution. Univariate and multivariate linear regression was used to examine the associations between right heart structure and peak exercise performance. A *p*-value < 0.05 was considered significant. JMP^®^ Pro 17.0.0 (622,752) (*JMP Statistical Software, Cary, NC,* United States) was used for statistical analyses.

## 3 Results

### 3.1 Study group characteristics

The study group had a mean BMI of 23.8 kg/m^2^ and age ranged from 18 to 58 years with a median of 29. Most cyclists were men (149; 71%). Over one-fourth of the participants described their health status as good and 49% as very good. All participants cycled regularly and it was their main sport activity. The median number of cycling training sessions per week was 4, with 6 h/week of cycle training. The minimum weekly cycling load was 2 h of training (17 participants, 8%). The mean value of MET minutes was 3,182. According to the IPAQ, most participants (55%) were classified in the high physical activity category, 35% in the moderate intensity category and 10% in the low intensity category. Apart from cycling, other sports included running (38%), gym (24%), swimming (17%), yoga (10%), CrossFit (7%), team sports (9%), others (31%).

Men had a higher median BMI of over 3 kg/m^2^ compared to women (24.5 *versus* 21.3 kg/m^2^). No other basic parameters differed between the sexes. Summary and comparisons of clinical characteristics in men and women are provided in Table 1.

**TABLE 1:** Study group characteristics including results of echocardiography and the cardiopulmonary exercise test (CPET) in men and women.

Parameter	Men (N = 149)		Women (N = 60)		
	Median	IQR	Median	IQR	*p*-value
BMI (kg/m^2^)	24.5	22.7–26.5	21.3	19.8–23.1	<0.001*
Age (years)	29	25–37	26	23–37	0.147
Cycling exercise days per week (days)	4	3–5	4	3–5	0.595
Weekly cycling training load (hours/week)	6	4–10	7	4–8	0.439
MET minutes (per week)	3400	1478–6124	2949	1446–5305	0.817
CPET duration (min)	10.7	9.8–11.6	10.0	8.9–11.1	0.087

	Men (Mean ± SD)		Women (Mean ± SD)		*p*-value
BASELINE					
IVSd (mm)	8.8 ± 1.4		7.4 ± 1.1		<0.001*
LVIDd (mm)	50.1 ± 5.1		44.5 ± 4.8		<0.001*
LVIDd BSA (mm/m^2^)	25.0 ± 2.5		26.5 ± 2.9		<0.001*
LVPWd (mm)	9.5 ± 1.2		8.4 ± 1.2		<0.001*
Biplane LVEF (Simpson) (%)	61.1 ± 3.6		61.8 ± 3.8		0.258
RVWT (mm)	6.1 ± 1.17		5.3 ± 1.1		<0.001*
RVIDd (mm)	32.3 ± 4.7		28.0 ± 4.4		<0.001*
RVD1 (mm)	40.9 ± 6.4		37.6 ± 5.8		<0.001*
RVD2 (mm)	28.7 ± 5.3		26.3 ± 5.1		<0.001*
RVD3 (mm)	92.2 ± 10.6		81.9 ± 8.5		<0.001*
RVAd (cm^2^)	25.6 ± 5.6		20.4 ± 4.8		<0.001*
RVAs (cm^2^)	13.6 ± 3.4		10.6 ± 2.9		0.177
LAV (mL)	57.0 ± 13.6		46.1 ± 12.9		<0.001*
LAVI (mL/m^2^)	28.4 ± 6.2		27.3 ± 639		0.290
RAV (mL)	48.4 ± 13.1		38.3 ± 11.6		<0.001*
RAVI (mL/m^2^)	24.1 ± 6.5		22.7 ± 6.2		0.145
BF (breaths/min)	15.3 ± 4.3		17.4 ± 4.3		<0.001*
VE/VCO_2_	32.9 ± 4.6		33.9 ± 3.8		0.101
VE/VO_2_	29.8 ± 7.0		30.8 ± 6.9		0.384
HR (beats/min)	83.3 ± 15.3		81.9 ± 15.1		0.539
O_2_ pulse (mL/beat)	6.2 ± 2.0		4.4 ± 1.3		<0.001*
PetCO_2_ (mmHg)	43.4 ± 5.0		40.7 ± 3.9		<0.001*
PetO_2_ (mmHg)	151.5 ± 9.0		154.7 ± 7.1		<0.001*
RER	0.91 ± 0.14		0.90 ± 0.13		0.673
VCO_2_ (L/min)	0.45 ± 0.17		0.31 ± 0.09		<0.001*
VO_2_ (L/min)	0.51 ± 0.18		0.35 ± 0.09		<0.001*
VE (L/min)	16.5 ± 5.3		12.6 ± 3.4		<0.001*
TV (L)	1.13 ± 0.55		0.77 ± 0.33		<0.001*
PEAK EXERCISE					
BF (breaths/min)	51.7 ± 11.7		51.1 ± 9.0		0.733
VE/VCO_2_	32.1 ± 4.1		32.5 ± 3.5		0.430
VE/VO_2_	39.8 ± 5.8		39.7 ± 4.8		0.868
HR (beats/min)	184.1 ± 11.8		182.5 ± 10.9		0.334
Load (W)	339.3 ± 64.3		226.6 ± 46.3		<0.001*
O_2_ pulse (mL/beat)	20.6 ± 3.4		13.9 ± 2.8		<0.001*
PetCO_2_ (mmHg)	46.0 ± 5.6		44.3 ± 4.5		0.018*
PetO_2_ (mmHg)	159.2 ± 5.9		160.5 ± 4.7		0.0926
RER	1.24 ± 0.07		1.21 ± 0.07		0.090
VCO_2_ (L/min)	4.68 ± 0.75		3.05 ± 0.54		<0.001*
VO_2_ (L/min)	3.79 ± 0.60		2.52 ± 0.46		<0.001*
VE (L/min)	157.4 ± 31.8		105.5 ± 18.6		<0.001*
TV (L)	3.19 ± 0.48		2.18 ± 0.37		<0.001*

Table 1 shows the study group characteristics for men and women. General characteristics are presented in the first section. In the second section results are shown from resting transthoracic echocardiography and cardiopulmonary exercise testing at baseline. The third section shows results from cardiopulmonary exercise testing at peak exercise. The last column shows the comparison *p*-value between men and women for all parameters. Values with asterisks represent *p* < 0.05.

Abbreviations: BF, breathing frequency; BMI, body mass index; CPET, cardiopulmonary exercise test; HR, heart rate; IVSd, diameter of intraventricular septum in diastole; LAV, end-systolic volume of left atrium (biplane measurement); LAVI, end-systolic volume of left atrium indexed to body surface area (biplane measurement); LVIDd, left ventricular internal diameter in diastole; LVIDd BSA, left ventricular internal diameter in diastole indexed to body surface area; LVPWd, left ventricular posterior wall diameter in diastole; LVEF, left ventricle ejection fraction; MET, the metabolic equivalent of task; O_2_ pulse, the ratio of VO_2_ to HR; PetCO_2_, the end-tidal carbon dioxide tension; PetO_2_, the end-tidal oxygen tension; RAV, end-systolic volume of right atrium; RAVI, end-systolic volume of right atrium indexed to body surface area (from 4-chamber view); RER, respiratory exchange ratio; RVAd, right ventricle area in diastole; RVAdI BSA, right ventricle area in diastole indexed to body surface area; RVAs, right ventricle area in systole; RVAsI BSA, right ventricle area in systole indexed to body surface area; RVD1, basal right ventricle dimension; RVD2, mid-cavity right ventricle dimension; RVD3, right ventricle longitudinal dimension; RVIDd, right ventricle internal diameter in diastole; RVWT, right ventricle wall thickness; SD, standard deviation; TV, tidal volume; VCO_2_, the volume of produced CO_2_; VE, minute ventilation; VE/VCO_2_, ventilatory equivalent for carbon dioxide; VE/VO_2_, ventilatory equivalent for oxygen; VO_2_, the volume of consumed O_2_.

### 3.2 Echocardiography and CPET

The mean values of most TTE parameters were in the normal range in both sexes (Table 1), except for RVD3 which was slightly elevated. Women had smaller chamber dimensions and thinner walls of RV and LV. Although the LA and RA volumes were smaller in women, after indexing to BSA, these differences disappeared.

Baseline and peak CPET results are presented in Table 1, additional timepoints (VT1 and VT2) are shown in Supplementary Table S2. Both groups performed adequate intense exercise reaching an RER (respiratory exchange ratio) of 1.24 in men and 1.21 in women. Compared to men, women had lower mean values of maximal load, peak O_2_ pulse, RER, VCO_2_, VO_2_, VO_2_/kg, VE (minute ventilation) and TV. BF (breathing frequency) and PetO_2_ were higher in women at rest, VT1 and VT2 but comparable to men at peak exercise. HR and RER were similar at rest and during the whole exercise. The ventilatory equivalents (VE/VCO_2_ and VE/VO_2_) were higher in women at VT1 and VT2 and similar at rest and peak exercise. Load, O_2_ pulse, PetCO_2_, VCO_2_, VO_2_, VO_2_/kg, VE and TV were lower in women at rest and during all exercise time points (VT1, VT2, peak).

### 3.3 Associations between peak CPET parameters and right heart structural parameters from ECHO in the sex subgroups

Table 2 shows the results of univariate linear regression for the peak HR, load, O_2_ pulse, and VO_2_ as a function of the correlations between RVD1, RVD3, RAVI and right ventricle wall thickness performed separately for men and women. In women, only RAVI was associated with exercise capacity indices such as BF, HR, load, O_2_ pulse, VCO_2_, VE, and VO_2_. Contrarily, in men, only RVD1 and RVD3 were associated with O_2_ pulse, load, VO_2_, VE and TV. RV wall thickness was not associated with any of the measured peak exercise capacity parameters in either men or women. These findings suggest that sex differences exist in the relationship between right heart structure and exercise performance in amateur cyclists.

**TABLE 2:** Associations between peak CPET parameters and right heart structural parameters from ECHO in the sex subgroups.

		Men			Women		
dependent	independent	slope	SE	*p*-value	slope	SE	*p*-value
HR peak	RAVI	−0.0607	0.1482	0.683	−0.5746	0.2237	0.013*
Load peak	RAVI	2.1061	0.8266	0.012*	3.0282	0.9072	0.002*
O_2_ pulse peak	RAVI	0.0852	0.0450	0.061	0.2058	0.0534	0.003*
VO_2_ peak	RAVI	0.0142	0.0078	0.073	0.0278	0.0093	0.004*
HR peak	RVD1	−0.2127	0.1495	0.157	−0.4192	0.2456	0.093
Load peak	RVD1	4.1611	0.7833	<0.001*	2.0725	1.0214	0.047*
O_2_ pulse peak	RVD1	0.2105	0.0429	<0.001*	0.1277	0.0617	0.043*
VO_2_ peak	RVD1	0.0343	0.0075	<0.001*	0.0164	0.0104	0.121
HR peak	RVD3	−0.1942	0.0904	0.034*	−0.1678	0.1707	0.330
Load peak	RVD3	2.2719	0.4868	<0.001*	1.5731	0.6921	0.027*
O_2_ pulse peak	RVD3	0.1343	0.0259	<0.001*	0.1139	0.0410	0.007*
VO_2_ peak	RVD3	0.0206	0.0046	<0.001*	0.0173	0.0069	0.015*
HR peak	RVWT	0.5467	0.8283	0.510	−0.3113	1.3286	0.816
Load peak	RVWT	−5.1487	4.6971	0.275	4.3208	5.5509	0.440
O_2_ pulse peak	RVWT	−0.1555	0.2540	0.542	0.4668	0.3321	0.170
VO_2_ peak	RVWT	−0.0202	0.0442	0.648	0.0760	0.0552	0.174

Table 2 shows the results of statistical analysis of associations between cardiopulmonary exercise testing parameters and right heart structural parameters in sex groups. Significant correlations are found for RAVI (end-systolic volume of right atrium indexed to body surface area, from 4-chamber view), RVD1 (basal right ventricle dimension) and RVD3 (right ventricle longitudinal dimension). No significant correlations were found for RVWT (right ventricle wall thickness). Values with asterisks represent *p* < 0.05.

Abbreviations: BF, breathing frequency; HR, heart rate; O_2_ pulse, the ratio of VO_2_ to HR; RAVI, end-systolic volume of right atrium indexed to body surface area (from 4-chamber view); RER, respiratory exchange ratio; RVD1, basal right ventricle dimension; RVD3, right ventricle longitudinal dimension; RVWT, right ventricle wall thickness; SE, standard error; TV, tidal volume; VCO_2_, the volume of produced CO_2_; VE, minute ventilation; VO_2_, the volume of consumed O_2_.

Data on the remaining RV structural parameters not shown in Table 2 and their association with peak exercise CPET parameters are presented in Supplementary Table S3.

### 3.4 Exercise performance prediction model: RVD1, RVD3, RAVI

**FIGURE 2:**
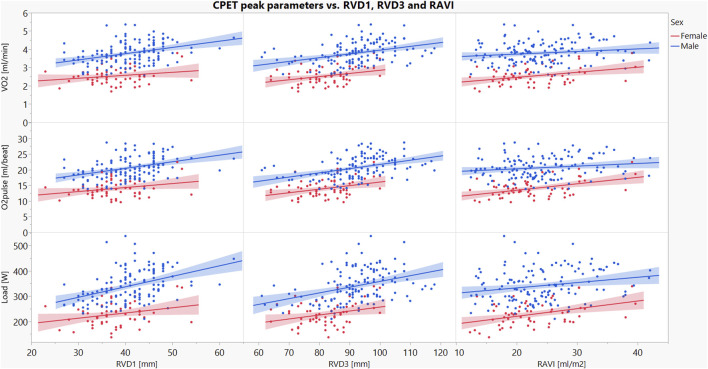
Linear regression of the relationships between RVD1, RVD3 or RAVI with peak load, VO_2_ and O_2_ pulse in male and female recreational cyclists. Red dots and shades represent women, and blue dots and shades represent men. The figure shows the associations between peak exercise performance parameters (load, O_2_ pulse, VO_2_) and right heart structural parameters (RVD1, RVD3 and RAVI). Abbreviations: O_2_ pulse–the ratio of VO_2_ to HR; RAVI–end-systolic volume of right atrium indexed to body surface area (from 4-chamber view); RVD1–basal right ventricle dimension; RVD3–right ventricle longitudinal dimension; VO_2_–the volume of consumed O_2_; O_2_ pulse–the ratio of VO_2_ to HR.

Multivariate linear regression models examined how RVD1, RVD3 and RAVI together predicted peak exercise capacity indices in men and women (Table 3; Figure 2). A higher RAVI predicted a better peak VO_2_, load and O_2_ pulse in women, with higher RVD1 and RVD3 predicting a better peak VO_2_, load and O_2_ pulse in men. None of the echocardiographic parameters were associated with peak HR in either sex. These findings suggest that there are sex differences in the determinants of exercise capacity in amateur cyclists.

**TABLE 3:** Exercise performance prediction model: RVD1, RVD3, RAVI.

		Men			Women		
dependent	independent	slope	SE	*p*-value	slope	SE	*p*-value
VO_2_ peak	RVD1	0.0212	0.0093	0.024*	−0.001	0.0113	0.990
	RVD3	0.0139	0.0053	0.010*	0.0115	0.0077	0.139
	RAVI	0.0057	0.0079	0.473	0.0221	0.0103	0.037*
Load peak	RVD1	2.5964	0.9617	0.008*	0.5771	1.1185	0.608
	RVD3	1.4347	0.5462	0.010*	0.7536	0.7556	0.323
	RAVI	1.0446	0.8185	0.204	2.4380	1.0162	0.020*
O_2_ pulse peak	RVD1	0.1215	0.0521	0.021*	0.0171	0.0653	0.795
	RVD3	0.0958	0.0296	0.002*	0.0644	0.0441	0.150
	RAVI	0.0339	0.0443	0.445	0.1673	0.0593	0.007*
HR peak	RVD1	−0.0136	0.1592	0.932	−0.138	0.2825	0.631
	RVD3	−0.0628	0.1870	0.737	0.0324	0.1984	0.871
	RAVI	−0.1744	0.1062	0.103	−0.5112	0.2600	0.055

Table 3 shows the results of multivariate linear regression models for RAVI (end-systolic volume of right atrium indexed to body surface area, from 4-chamber view), RVD1 (basal right ventricle dimension) and RVD3 (right ventricle longitudinal dimension) for predicting peak cardiopulmonary exercise testing parameters in sex groups. In men RVD1 and RVD3 predict better exercise capacity parameters. In women RAVI, predicts better exercise capacity parameters. Values with asterisks represent *p* < 0.05.

Abbreviations: HR, heart rate; O_2_ pulse, the ratio of VO_2_ to HR; RAVI, end-systolic volume of right atrium indexed to body surface area; RVD1, basal right ventricle dimension; RVD3, right ventricle longitudinal dimension; SE, standard error VO_2_, the volume of consumed O_2_.

## 4 Discussion

Our study confirms prior research showing thicker RV and LV walls, and larger LV, RV, LA and RA cavities in amateur male cyclists. We also found significant associations between right heart dimensions and peak CPET values in amateur cyclists with different levels of training. More importantly, these associations differ by sex, a finding not previously reported. Better CPET parameters were predicted by larger RA volume only in women, while larger RVD1 and RVD3 predicted better performance only in men. Notably, RV wall thickness did not predict peak CPET indices in either sex.

Consistent with previous studies, (D’Ascenzi et al., 2017; Colombo and Finocchiaro, 2018; Marsh et al., 2021; St. Pierre et al., 2022), men in our study exhibited larger cardiac dimensions, including RA and LA volumes, LV mass, LV and RV end-diastolic diameters and wall thicknesses. Additionally, the sex differences in RA and LA volumes are no longer significant after indexing them to BSA. This finding aligns with previous studies (D’Ascenzi et al., 2017; Conti et al., 2021) and suggests that the sex differences for the atria, but not the ventricles, are determined by body size.

The male cyclists in our study achieved higher peak workloads, O_2_ pulse, VCO_2_, VO_2_, VE and TV during CPET to exhaustion, consistent with previous studies (Bassett et al., 2020; Conti et al., 2021; St. Pierre et al., 2022). However, our observations suggest sex differences in the associations between right heart structural changes and exercise capacity in amateur cyclists. Peak CPET parameters are associated with RV diameters in men but RA volumes in women.

### 4.1 Adaptation of the right heart to exercise

In resting conditions, the thinner and smaller RV generates lower pressures than the LV to eject similar stroke volume. This is due to lower resistance (afterload) in the pulmonary circulation compared to the systemic circulation. However, during exercise, the same increase in preload and the amount of blood pumped during exercise has different effects on each ventricle. The RV afterload increases disproportionally to LV afterload, leading to a greater relative RV wall stress than the LV experiences (La Gerche et al., 2011). The combination of increased preload and thinner walls contributes to a stronger relative RV dilation than the LV.

Endurance athletes have larger RV and RA dimensions than sedentary controls (Erol and Karakelleoglu, 2002), with the RA dilation responding to endurance exercise, its duration and intensity, and the RV basal diameter (D’Ascenzi et al., 2018). The RA size is positively correlated with age, suggesting that the total time spent training affects right heart adaptation (Conti et al., 2021). The intensity of training also shows a positive correlation with RV dimensions, area and volume (Conti et al., 2021; Ramcharan et al., 2023).

### 4.2 Sex differences in adaptation of the heart to exercise

The overall response of the cardiovascular system to exercise and training is similar in men and women. Men have greater LV mass and RV diameters than women (Sanz-de La Garza et al., 2017; D’Ascenzi et al., 2018; Conti et al., 2021). Endurance training leads to similar structural remodeling of both ventricles in both sexes (Sanz-de La Garza et al., 2017), resulting in the dilation of both ventricles in male and female athletes. However, the RV and RA dimensions remain larger in men (Sanz-de La Garza et al., 2017; Conti et al., 2021).

We report that peak VO_2_, O_2_ pulse and load were associated with larger RV diameters in men but more dilated RA in women. It suggests that in amateur cyclists, different structural characteristics of the right heart are related to better exercise capacity.

One possibility is that adaptation to endurance exercise might follow a different pattern in men and women. This could be due to differences in body size, muscle mass and how the cardiovascular system handles increased blood flow during exercise. Women have better RV systolic function than men. However, men have higher RV mass and volumes and lower RV ejection fraction (Keen et al., 2021). Superior RV function in women persists even after multivariate adjustment for LV function and body size (Keen et al., 2021). Total pulmonary resistance and pulmonary arterial elastance are higher at rest and exercise in women (Sless et al., 2023). Women with higher exercise capacity, as measured by CPET, may have a larger RA. This potentially results from thinner RA walls, which can better adapt to high hemodynamic stress caused by increased blood returning from systemic circulation. On the other hand, RV dimensions in women do not predict better exercise performance, unlike men. Men’s larger RV dimensions likely stem form accommodating a greater blood volume for lung delivery during exercise.

Men and women probably have significant differences in exercise-induced blood flow through the pulmonary circulation. Men typically have larger bodies and muscle mass, which results in more circulating blood during exercise and a higher venous return to the right heart. If so, a larger volume of blood pumped by the right ventricle could translate into a relatively higher vascular resistance within the pulmonary circulation. Consequently, the afterload on the RV might be disproportionally higher in men than women during exercise.

Data on sex differences in exercise-induced pulmonary vascular resistance or right ventricle afterload are currently scarce. For instance, during CPET men with HFpEF have a more pronounced increase in RV afterload and they fail to increase RV contractility (Singh et al., 2021). Optimal medical therapy in pulmonary arterial hypertension leads to significant decreases in mean pulmonary arterial pressure and pulmonary vascular resistance in both sexes. However, only females have an improvement in right ventricular end-diastolic and right atrial pressures, cardiac index and mixed venous oxygen saturation (Kozu et al., 2018). In patients with pulmonary hypertension the pulmonary vascular resistance and RV ejection fraction are comparable between men and women at baseline (Shelburne et al., 2024). Also a similar reduction in pulmonary vascular resistance in both sexes was shown. However, men had a worse transplant-free survival and only women showed an increase in RV ejection fraction (Shelburne et al., 2024). Men with arrhythmogenic RV cardiomyopathy have lower RV ejection fraction and higher RV end-diastolic volume than women (Keen et al., 2021). Some of these observations and other factors related to sex differences, which we do not yet understand or account for, might also contribute. This issue deserves more detailed studies.

### 4.3 The right ventricle shape in adaptation to endurance exercise

Bernardino et al. found that endurance athletes had a more spherical RV shape than sedentary controls using MRI (Bernardino et al., 2020). The RV’s regional geometry change, characterized by an increased diameter of the apex and outflow regions, was associated with peak O_2_ pulse (Bernardino et al., 2020). We noticed similar associations between RV diameters and peak O_2_ pulse in univariate analysis in both sexes. However, in a multivariate analysis RVD1 and RVD3, but not RAVI, contributed significantly to peak CPET parameters only in men. The RV mid-cavity diameter was not correlated with O_2_ pulse in either sex. We therefore hypothesize that RVD1 and RVD3 could be more affected by the effects of endurance training than RVD2.

### 4.4 Right atrial enlargement in women cyclists

Men have a larger RA area compared to women (Sanz-de La Garza et al., 2017; D’Ascenzi et al., 2018; Conti et al., 2021), but standardization for height, greatly attenuates these differences (Keller et al., 2021). Our results are consistent with these studies. Typically, RA enlargement is caused by increased preload, usually secondary to tricuspid regurgitation (Nemoto et al., 2017). However, our subjects lacked significant tricuspid insufficiency at rest. Furthermore, men are typically expected to exhibit greater atrial enlargement due to a larger RV and tricuspid annulus, potentially predisposing them to more severe tricuspid regurgitation. Interestingly, our findings showed that women had greater atrial enlargement despite having a smaller RV, including RVD1. Our findings are probably the first to demonstrate that better cycling performance is associated with larger RV in men but more dilated RA in women.

### 4.5 Potential causes of differences in right heart morphology between men and women

The human heart undergoes continuous changes in size, weight, structure and function throughout life. Repeated training, especially intensive and prolonged exercise, leads to exercise-induced muscle damage, including the myocardium (Liu et al., 2023). All of these factors affect both the left and right heart but have different consequences in men and women.

Numerous sex-related factors may influence right heart remodeling, including differences in body size, muscle mass, heart dimensions, coronary artery size, coronary blood flow, metabolic efficiency, vascular remodeling, environmental factors (diet, time spent training, social responsibilities) and genetic factors such as chromosomes, genes, and sex hormones (Beale et al., 2018). Female and male hearts are similar in size at younger ages. A significant divergence in the heart’s mass and size begins during puberty (de Simone et al., 1995). Adult female hearts weigh between 230 and 280 g, about 1/4 lighter than male hearts, which range between 280 and 340 g (St. Pierre et al., 2022).

Differences in training volumes could also account for the variation in cardiac adaptation. Men often have higher training volumes. For example, male marathon runners reported greater weekly running distances but shorter training time, however, they reached 10% higher running velocity (Haugen et al., 2022). Conversely, some studies suggest that in certain endurance sports like triathlon and ultra-endurance events, women may be able to achieve the same or greater training volumes than men (Haugen et al., 2022). These observations may be caused by women’s better relative endurance and resistance to fatigue. Nevertheless, top female athletes achieve poorer results than top men during the same competitions.

### 4.6 Differences from other investigations and study novelty

Unlike many previous studies focuses on professional athletes, our study uniquely focuses on amateur cyclists. We show that some structural changes in the right heart typical for professional endurance athletes also apply to amateur cyclists. Our findings demonstrate that cycling performance is better predicted by RVD1 and RVD3 in men while RAVI is more relevant in women. While our study partially confirms observations from previous research (e.g., sex differences in cardiac sizes and CPET parameters), it also presents novel findings due to its focus on amateur cyclists with diverse training volumes and exercise capacities.

### 4.7 Study limitations

Due to its cross-sectional design, our study cannot definitively determine whether observed associations reflect causal relationships. However, the findings provide a strong foundation for future research exploring potential causal mechanisms. While we measured the right heart structure at rest, assessing it during maximal exercise proved challenging due to limitations of echocardiography, specifically movement and breathing-related artifacts. Additionally, due to word and page limits, we were unable to include functional indices of the right heart in this study; these will be addressed in a separate investigation.

By recruiting a varied group of cyclists with different levels of training (2–14 h/week), we achieved a more representative sample of amateur cyclists. The heterogeneity improves the applicability of our findings to a more general population. Cycling was the primary sport for participants, although many also participated in other sports, including power sports (common), other endurance sports (common), team sports (mostly men), yoga (mostly women), and skill-based activities (some). The training load of other sports was typically lower and less important to the athletes. We focused exclusively on cyclists of European ethnicity. Recruitment was based on consecutive volunteers who responded to a public call, resulting in an imbalanced sex distribution (more men than women enrolled). However, this reflects the actual sex distribution in amateur cycling. While our sample of cyclists at various levels of exercise capacity and training volume strengthens generalizability, limitations in ethnicity and sex distribution must be considered when interpreting and applying our findings.

### 4.8 Perspective

Our findings suggest that regardless of the underlying mechanisms, there are sex differences in the relationships between right heart structure and peak exercise capacity. For men, monitoring the RV size might be relevant when assessing exercise capacity. In contrast, this information appears to have no such importance in women. However, for female but, not male cyclists, considering RA size could be important.

Echocardiographic measurements hold promise for optimizing training protocols in cyclists. By assessing RV and RA sizes, tailored training regimens could be designed. However, this approach remains untested and is likely more applicable to professional cyclists due to associated costs. Preselecting top male cyclists with larger RV for endurance events and top female cyclists with more dilated RA for peak performance is speculative, but intriguing. Generalizing these findings to other endurance sports or non-European athletes requires further investigation. Additionally, exploring three-dimensional echocardiography could yield more precise mechanisms underlying these sex-based differences in right heart and their impact on exercise performance in amateur cyclists. It should also be explored whether a dose-effect relationship exists between right heart structural parameters and CPET performance.

## 5 Conclusion

We investigated the relationship between right heart structure and exercise capacity in amateur cyclists with varying training levels, as well as sex differences in these relationships. Men had larger overall cardiac dimensions and achieved higher peak performance on all CPET parameters compared to women. We found significant associations between right heart dimensions and peak CPET parameters in both men and women. However, these associations differed by sex. In men, larger RVD1 and RVD3 were associated with better exercise performance. In contrast, in women, the RAVI predicted higher peak CPET values. RV wall thickness was not a predictor of peak CPET parameters in either sex.

Our findings suggest that amateur male and female cyclists exhibit distinct patterns of right heart structural changes, which are linked to exercise capacity. These findings encourage further research to explore causal mechanisms, as well as studying a more diverse population.

## Data Availability

The original contributions presented in the study are included in the article/Supplementary Material, further inquiries can be directed to the corresponding author.

## References

[B1] Bassett A. J. Ahlmen A. Rosendorf J. M. Romeo A. A. Erickson B. J. Bishop M. E. (2020). The Biology of Sex and Sport. JBJS Rev. 8, e0140. 10.2106/JBJS.RVW.19.00140 32224635

[B2] Beale A. L. Meyer P. Marwick T. H. Lam C. S. P. Kaye D. M. (2018). Sex Differences in Cardiovascular Pathophysiology: Why Women Are Overrepresented in Heart Failure With Preserved Ejection Fraction. Circulation 138, 198–205. 10.1161/CIRCULATIONAHA.118.034271 29986961

[B3] Bernardino G. Sanz De La Garza M. Domenech-Ximenos B. Prat-Gonzàlez S. Perea R. J. Blanco I. (2020). Three-Dimensional Regional Bi-Ventricular Shape Remodeling is Associated with Exercise Capacity in Endurance Athletes. Eur. J. Appl. Physiol. 120, 1227–1235. 10.1007/s00421-020-04335-3 32130484

[B4] Churchill T. W. Petek B. J. Wasfy M. M. Guseh J. S. Weiner R. B. Singh T. K. (2021). Cardiac Structure and Function in Elite Female and Male Soccer Players. JAMA Cardiol. 6, 316–325. 10.1001/jamacardio.2020.6088 33263734 PMC7711565

[B5] Colombo C. S. S. S. Finocchiaro G. (2018). The Female Athlete’s Heart: Facts and Fallacies. Curr. Treat. Options Cardio Med. 20, 101. 10.1007/s11936-018-0699-7 PMC6223714 30390143

[B6] Conti V. Migliorini F. Pilone M. Barriopedro M. I. Ramos-Álvarez J. J. Montero F. J. C. (2021). Right Heart Exercise-Training-Adaptation and Remodelling In Endurance Athletes. Sci. Rep. 11, 22532. 10.1038/s41598-021-02028-1 34795399 PMC8602371

[B7] D’Ascenzi F. Anselmi F. Focardi M. Mondillo S. (2018). Atrial Enlargement in the Athlete’s Heart: Assessment of Atrial Function May Help Distinguish Adaptive from Pathologic Remodeling. J. Am. Soc. Echocardiogr. 31, 148–157. 10.1016/j.echo.2017.11.009 29246514

[B8] D’Ascenzi F. Cavigli L. Marchese A. Taddeucci S. Cappelli E. Roselli A. (2024). Electrical and Structural Remodelling in Female Athlete’s Heart: A Comparative Study in Women vs Men Athletes and Controls. Int. J. Cardiol. 400, 131808. 10.1016/j.ijcard.2024.131808 38262482

[B9] D’Ascenzi F. Pelliccia A. Solari M. Piu P. Loiacono F. Anselmi F. (2017). Normative Reference Values of Right Heart in Competitive Athletes: A Systematic Review and Meta-Analysis. J. Am. Soc. Echocardiogr. 30, 845–858. 10.1016/j.echo.2017.06.013 28865556

[B10] de Simone G. Devereux R. B. Daniels S. R. Meyer R. A. (1995). Gender Differences in Left Ventricular Growth. Hypertension 26, 979–983. 10.1161/01.HYP.26.6.979 7490158

[B11] Erol M. K. Karakelleoglu S. (2002). Assessment of Right Heart Function in the Athlete’s Heart. Heart Vessels 16, 175–180. 10.1007/s003800200018 12181590

[B12] Gargani L. Pugliese N. R. De Biase N. Mazzola M. Agoston G. Arcopinto M. (2023). Exercise Stress Echocardiography of the Right Ventricle and Pulmonary Circulation. J. Am. Coll. Cardiol. 82, 1973–1985. 10.1016/j.jacc.2023.09.807 37968015

[B13] Guzik P. Więckowska B. (2023). Data Distribution Analysis – A Preliminary Approach to Quantitative Data in Biomedical Research. J. Med. Sci. 92, e869. 10.20883/medical.e869

[B14] Hagströmer M. Oja P. Sjöström M. (2006). The International Physical Activity Questionnaire (IPAQ): A Study of Concurrent and Construct Validity. Public Health Nutr. 9, 755–762. 10.1079/PHN2005898 16925881

[B15] Haugen T. Sandbakk Ø. Seiler S. Tønnessen E. (2022). The Training Characteristics of World-Class Distance Runners: An Integration of Scientific Literature and Results-Proven Practice. Sports Med. - Open 8, 46. 10.1186/s40798-022-00438-7 35362850 PMC8975965

[B16] Haykowsky M. J. Samuel T. J. Nelson M. D. La Gerche A. (2018). Athlete’s Heart: Is the Morganroth Hypothesis Obsolete? Heart, Lung Circulation 27, 1037–1041. 10.1016/j.hlc.2018.04.289 29773412

[B17] Hellsten Y. Nyberg M. (2015). “Cardiovascular Adaptations to Exercise Training,” in Comprehensive Physiology. Editor Terjung R. (New York, NY: Wiley), 1–32. 10.1002/cphy.c140080 26756625

[B18] Jones N. Burns A. T. Prior D. L. (2019). Echocardiographic Assessment of the Right Ventricle–State of the Art. Heart, Lung Circulation 28, 1339–1350. 10.1016/j.hlc.2019.04.016 31175016

[B19] Keen J. Prisco S. Z. Prins K. W. (2021). Sex Differences in Right Ventricular Dysfunction: Insights From the Bench to Bedside. Front. Physiol. 11, 623129. 10.3389/fphys.2020.623129 33536939 PMC7848185

[B20] Keller K. Sinning C. Schulz A. Jünger C. Schmitt V. H. Hahad O. (2021). Right Atrium Size in the General Population. Sci. Rep. 11, 22523. 10.1038/s41598-021-01968-y 34795353 PMC8602329

[B21] Kozu K. Sugimura K. Aoki T. Tatebe S. Yamamoto S. Yaoita N. (2018). Sex Differences in Hemodynamic Responses and Long-Term Survival to Optimal Medical Therapy in Patients with Pulmonary Arterial Hypertension. Heart Vessels 33, 939–947. 10.1007/s00380-018-1140-6 29441403 PMC6060798

[B22] La Gerche A. Heidbüchel H. Burns A. T. Mooney D. J. Taylor A. J. Pfluger H. B. (2011). Disproportionate Exercise Load and Remodeling of the Athlete’s Right Ventricle. Med. Sci. Sports Exerc. 43, 974–981. 10.1249/MSS.0b013e31820607a3 21085033

[B23] La Gerche A. Wasfy M. M. Brosnan M. J. Claessen G. Fatkin D. Heidbuchel H. (2022). The Athlete’s Heart—Challenges and Controversies: JACC Focus Seminar 4/4. J. Am. Coll. Cardiol. 80, 1346–1362. 10.1016/j.jacc.2022.07.014 36075838

[B24] Lewicka-Potocka Z. Dąbrowska-Kugacka A. Lewicka E. Kaleta A. M. Dorniak K. Daniłowicz-Szymanowicz L. (2019). The “Athlete’s Heart” Features in Amateur Male Marathon Runners. Cardiol. J. 28, 707–715. 10.5603/CJ.a2019.0110 PMC8428944 31909474

[B25] Liu C. Wu X. Vulugundam G. Gokulnath P. Li G. Xiao J. (2023). Exercise Promotes Tissue Regeneration: Mechanisms Involved and Therapeutic Scope. Sports Med. Open 9, 27. 10.1186/s40798-023-00573-9 37149504 PMC10164224

[B26] Marsh C. E. Thomas H. J. Naylor L. H. Dembo L. G. Green D. J. (2021). Sex Differences in Cardiac Adaptation to Distinct Modalities of Exercise: A Cardiac Magnetic Resonance Study. Med. Sci. Sports Exerc. 53, 2543–2552. 10.1249/MSS.0000000000002729 34138817

[B27] Mitchell C. Rahko P. S. Blauwet L. A. Canaday B. Finstuen J. A. Foster M. C. (2019). Guidelines for Performing a Comprehensive Transthoracic Echocardiographic Examination in Adults: Recommendations from the American Society of Echocardiography. J. Am. Soc. Echocardiogr. 32, 1–64. 10.1016/j.echo.2018.06.004 30282592

[B28] Morganroth J. Maron B. J. Henry W. L. Epstein S. E. (1975). Comparative Left Ventricular Dimensions in Trained Athletes. Ann. Intern Med. 82, 521–524. 10.7326/0003-4819-82-4-521 1119766

[B29] Nemoto N. Schwartz J. G. Lesser J. R. Pedersen W. D. Sorajja P. Garberich R. (2017). The Right Atrium and Tricuspid Annulus are Cardinal Structures in Tricuspid Regurgitation with or Without Pulmonary Hypertension. Int. J. Cardiol. 230, 171–174. 10.1016/j.ijcard.2016.11.075 27979575

[B30] Pritchard A. Burns P. Correia J. Jamieson P. Moxon P. Purvis J. (2021). ARTP Statement on Cardiopulmonary Exercise Testing 2021. BMJ Open Resp. Res. 8, e001121. 10.1136/bmjresp-2021-001121 PMC8593741 34782330

[B31] Qiu Y. Pan X. Chen Y. Xiao J. (2022). Hallmarks of Exercised Heart. J. Mol. Cell. Cardiol. 164, 126–135. 10.1016/j.yjmcc.2021.12.004 34914934

[B32] Ramcharan T. Edwards J. O’Driscoll J. Papadakis M. (2023). The Acute Impact of Endurance Exercise on Right Ventricular Structure and Function: A Systematic Review and Meta-analysis. Cardiol. Clin. 41, 25–34. 10.1016/j.ccl.2022.08.004 36368809

[B33] Sanz-de La Garza M. Giraldeau G. Marin J. Grazioli G. Esteve M. Gabrielli L. (2017). Influence of Gender on Right Ventricle Adaptation to Endurance Exercise: An Ultrasound Two-Dimensional Speckle-Tracking Stress Study. Eur. J. Appl. Physiol. 117, 389–396. 10.1007/s00421-017-3546-8 28150069

[B34] Sawicka-Gutaj N. Gruszczyński D. Guzik P. Mostowska A. Walkowiak J. (2022). Publication Ethics of Human Studies in the Light of the Declaration of Helsinki – A Mini-Review. J. Med. Sci. 91, e700. 10.20883/medical.e700

[B35] Shelburne N. J. Nian H. Beck G. J. Casanova N. G. Desai A. A. DuBrock H. M. (2024). Male Sex Is Associated With Worse Right Ventricular Function and Survival in Pulmonary Hypertension in the Redefining Pulmonary Hypertension Through Pulmonary Vascular Disease Phenomics Cohort. CHEST Pulm., 100046. 10.1016/j.chpulm.2024.100046 PMC11548889 39524046

[B36] Singh I. Oliveira R. K. F. Heerdt P. M. Pari R. Systrom D. M. Waxman A. B. (2021). Sex-Related Differences in Dynamic Right Ventricular-Pulmonary Vascular Coupling in Heart Failure with Preserved Ejection Fraction. Chest 159, 2402–2416. 10.1016/j.chest.2020.12.028 33388286

[B37] Sless R. T. Wright S. P. Bentley R. F. Valle F. H. Mak S. (2023). Sex Differences in Pulmonary and Systemic Vascular Function at Rest and During Exercise In Healthy Middle-Aged Adults. J. Hum. Hypertens. 37, 746–752. 10.1038/s41371-023-00822-0 36997720

[B38] St. Pierre S. R. Peirlinck M. Kuhl E. (2022). Sex Matters: A Comprehensive Comparison of Female and Male Hearts. Front. Physiology 13, 831179. 10.3389/fphys.2022.831179 PMC8980481 35392369

