# Supplementary material for: Sex differences in the associations between right heart structure and peak exercise capacity parameters in amateur cyclists
Source: Front Physiol. 2024 Jul 29;15:1427101. doi: 10.3389/fphys.2024.1427101 (PMC11318071; doi:10.3389/fphys.2024.1427101)
Supplement: Supplementary file 1 [file DataSheet1.docx]

**Supplementary Table 1. Parameters measured during cardiopulmonary exercise testing.**

| **Abbreviation** | **Full name of the parameter** |
| --- | --- |
| HR | Heart rate |
| VO_2_ | The volume of consumed O_2_ per minute |
| O_2_ pulse | The ratio of VO_2_ to HR |
| VCO_2_ | The volume of produced CO_2_ per minute |
| VE | Minute ventilation |
| TV | The tidal volume of a single breath |
| BF | Breathing frequency |
| VE/VCO_2_ | The ventilatory equivalent for CO_2_ |
| VE/VO_2_ | The ventilatory equivalent for O_2_ |
| RER | Respiratory exchange ratio |
| PETO_2_ | The end-tidal O_2_ tension in exhaled air |
| PETCO_2_ | The end-tidal CO_2_ tension in exhaled air |

Supplementary Table 1 collects all analyzed parameters from the cardiopulmonary exercise test and shows the used abbreviations and full names of the parameters.

**Supplementary Table 2 Parameters measured during cardiopulmonary exercise testing.**

| **Parameter** | **Men (N=149)**  **(Mean ± SD)** | **Women (N=60)**  **(Mean ± SD)** | ***p* value** |
| --- | --- | --- | --- |
| **VT1** | | | |
| BF (breaths/min) | 26.5±5.2 | 29.0±5.5 | <0.001* |
| VE/VCO_2_ | 25.2±2.6 | 26.5±2.6 | <0.001* |
| VE/VO_2_ | 24.5±2.9 | 25.5±3.2 | 0.044* |
| HR (beats/min) | 151.4±13.6 | 154.2±13.6 | 0.185 |
| Load (W) | 205.3±50.3 | 128.8±35.3 | <0.001* |
| O_2_ pulse (mL/beat) | 17.9±3.6 | 11.9±2.5 | <0.001* |
| PetCO_2_ (mmHg) | 57.0±5.0 | 53.4±4.9 | <0.001* |
| PetO_2_ (mmHg) | 139.2±6.4 | 142.9±6.7 | <0.001* |
| RER | 0.97±0.06 | 0.96±0.06 | 0.170 |
| VCO_2_ (L/min) | 2.62±0.51 | 1.74±0.32 | <0.001* |
| VO_2_ (L/min) | 2.70±0.55 | 1.83±0.37 | <0.001* |
| VE (L/min) | 69.7±15.6 | 49.9±9.6 | <0.001* |
| TV (L) | 2.77±0.51 | 1.84±0.36 | <0.001* |
| **VT2** | | | |
| BF (breaths/min) | 32.7±6.0 | 36.6±7.9 | <0.001* |
| VE/VCO_2_ | 26.1±2.9 | 27.7±2.9 | <0.001* |
| VE/VO_2_ | 28.4±3.5 | 30.3±3.4 | <0.001* |
| HR (beats/min) | 170.5±12.4 | 172.3±11.6 | 0.345 |
| Load (W) | 279.4±57.9 | 184.7±40.8 | <0.001* |
| O_2_ pulse (mL/beat) | 19.9±3.7 | 13.3±2.8 | <0.001* |
| PetCO_2_ (mmHg) | 55.3±5.4 | 51.5±5.1 | <0.001* |
| PetO_2_ (mmHg) | 145.8±5.9 | 150.1±5.5 | <0.001* |
| RER | 1.09±0.05 | 1.09±0.04 | 0.432 |
| VCO_2_ (L/min) | 3.69±0.67 | 2.50±0.46 | <0.001* |
| VO_2_ (L/min) | 3.39±0.63 | 2.29±0.45 | <0.001* |
| VE (L/min) | 100.8±21.6 | 73.6±13.8 | <0.001* |
| TV (L) | 3.22±0.53 | 2.14±0.40 | <0.001* |

Supplementary Table 2 shows results from cardiopulmonary exercise testing at ventilatory thresholds 1 and 2. The last column shows the comparison *p*-value between men and women for all parameters. Values with asterisks represent *p*<0.05

Abbreviations: BF – breathing frequency; HR – heart rate; PetCO_2_ – the end-tidal carbon dioxide tension; PetO_2_ – the end-tidal oxygen tension; RER – respiratory exchange ratio; SD – standard deviation. TV – tidal volume; VCO_2_ – the volume of produced CO_2_; VE – minute ventilation; VE/VCO_2_ – ventilatory equivalent for carbon dioxide; VE/VO_2_ – ventilatory equivalent for oxygen; VO_2_ – the volume of consumed O_2_; VT1 – ventilatory threshold 1; VT2 – ventilatory threshold 2;

## Supplementary Table 3 Associations between peak CPET parameters and RV structural parameters from ECHO in the sex subgroups.

|  |  | Men | | | Women | | |
| --- | --- | --- | --- | --- | --- | --- | --- |
| dependent | independent | slope | SE | P value | slope | SE | P value |
| RAVI | BF PEAK | -0.2868 | 0.1508 | 0.0592 | 0.3859 | 0.1881 | 0.0449* |
| RAVI | RER PEAK | -0.0010 | 0.0009 | 0.2531 | -0.0027 | 0.0016 | 0.1089 |
| RAVI | VCO_2_ PEAK | 0.0116 | 0.0098 | 0.2405 | 0.0292 | 0.0110 | 0.0100* |
| RAVI | VE PEAK | -0.5644 | 0.4105 | 0.1714 | 1.1381 | 0.3742 | 0.0036* |
| RAVI | TV PEAK | 0.0095 | 0.0065 | 0.1455 | 0.0060 | 0.0079 | 0.4460 |
| RVD1 | BF PEAK | 0.1319 | 0.1556 | 0.3979 | 0.0492 | 0.2077 | 0.8135 |
| RVD1 | RER PEAK | -0.0015 | 0.0009 | 0.0875 | -0.0018 | 0.0018 | 0.3174 |
| RVD1 | VCO_2_ PEAK | 0.0358 | 0.0095 | 0.0003* | 0.0175 | 0.0122 | 0.1558 |
| RVD1 | VE PEAK | 1.0230 | 0.4127 | 0.0144* | 0.6625 | 0.4211 | 0.1213 |
| RVD1 | TV PEAK | 0.0159 | 0.0065 | 0.0160* | 0.0133 | 0.0082 | 0.1127 |
| RVD3 | BF PEAK | 0.1108 | 0.0944 | 0.2429 | 0.1344 | 0.1409 | 0.3440 |
| RVD3 | RER PEAK | -0.0010 | 0.0005 | 0.0705 | -0.0020 | 0.0012 | 0.0958 |
| RVD3 | VCO_2_ PEAK | 0.0212 | 0.0058 | 0.0004* | 0.0144 | 0.0082 | 0.0870 |
| RVD3 | VE PEAK | 0.8981 | 0.2453 | 0.0004* | 0.4437 | 0.2881 | 0.1291 |
| RVD3 | TV PEAK | 0.0129 | 0.0039 | 0.0011* | 0.0067 | 0.0057 | 0.2418 |
| RAVol | BF PEAK | -0.1666 | 0.0751 | 0.0281* | 0.2018 | 0.1009 | 0.0504 |
| RAVol | HR PEAK | -0.0909 | 0.0738 | 0.2199 | -0.3202 | 0.1192 | 0.0095* |
| RAVol | Load PEAK | 1.4439 | 0.4047 | 0.0005* | 2.0562 | 0.4557 | <0.0001* |
| RAVol | O_2_ pulse PEAK | 0.0808 | 0.0217 | 0.0003* | 0.1413 | 0.0261 | <0.0001* |
| RAVol | RER PEAK | -0.0010 | 0.0004 | 0.0270* | -0.0017 | 0.0009 | 0.0602 |
| RAVol | VCO_2_ PEAK | 0.0113 | 0.0048 | 0.0209* | 0.0213 | 0.0055 | 0.0003* |
| RAVol | VE PEAK | -0.0664 | 0.2067 | 0.7484 | 0.7730 | 0.1901 | 0.0002* |
| RAVol | VO_2_ PEAK | 0.0129 | 0.0038 | 0.0009* | 0.0202 | 0.0046 | <0.0001* |
| RAVol | TV PEAK | 0.0106 | 0.0031 | 0.0010* | 0.0068 | 0.0041 | 0.1058 |
| RVAd | BF PEAK | 0.1589 | 0.1804 | 0.3801 | 0.3154 | 0.2507 | 0.2137 |
| RVAd | HR PEAK | -0.3378 | 0.1727 | 0.0526 | -0.5480 | 0.2995 | 0.0726 |
| RVAd | Load PEAK | 3.9792 | 0.9395 | <0.0001* | 2.8405 | 1.2382 | 0.0256* |
| RVAd | O_2_ pulse PEAK | 0.2549 | 0.0495 | <0.0001* | 0.1964 | 0.0738 | 0.0101* |
| RVAd | RER PEAK | -0.0032 | 0.0010 | 0.0022* | -0.0023 | 0.0021 | 0.2921 |
| RVAd | VCO_2_ PEAK | 0.0387 | 0.0112 | 0.0007* | 0.0258 | 0.0148 | 0.0858 |
| RVAd | VE PEAK | 1.5548 | 0.4717 | 0.0013* | 1.0774 | 0.5065 | 0.0378 |
| RVAd | VO_2_ PEAK | 0.0401 | 0.0087 | <0.0001* | 0.0261 | 0.0125 | 0.0419 |
| RVAd | TV PEAK | 0.0248 | 0.0074 | 0.0011* | 0.0140 | 0.0102 | 0.1731 |
| RVAdI | BF PEAK | 0.5879 | 0.3958 | 0.1398 | 0.4919 | 0.4075 | 0.2325 |
| RVAdI | HR PEAK | -0.5947 | 0.3830 | 0.1228 | -0.7481 | 0.4905 | 0.1328 |
| RVAdI | Load PEAK | 8.2994 | 2.0871 | 0.0001* | 1.6049 | 2.0916 | 0.4461 |
| RVAdI | O_2_ pulse PEAK | 0.4335 | 0.1134 | 0.0002* | 0.1241 | 0.1261 | 0.3292 |
| RVAdI | RER PEAK | -0.0033 | 0.0023 | 0.1600 | -0.0018 | 0.0035 | 0.6064 |
| RVAdI | VCO_2_ PEAK | 0.0732 | 0.0250 | 0.0039* | 0.0070 | 0.0246 | 0.7762 |
| RVAdI | VE PEAK | 2.9304 | 1.0517 | 0.0061* | 0.7840 | 0.8485 | 0.3594 |
| RVAdI | VO_2_ PEAK | 0.0670 | 0.0199 | 0.0010* | 0.0099 | 0.0211 | 0.6394 |
| RVAdI | TV PEAK | 0.0288 | 0.0168 | 0.0891 | 0.0038 | 0.0167 | 0.8221 |
| RVAs | BF PEAK | 0.3982 | 0.2909 | 0.1734 | 0.5450 | 0.4136 | 0.1929 |
| RVAs | HR PEAK | -0.4381 | 0.2811 | 0.1215 | -0.8723 | 0.4957 | 0.0839 |
| RVAs | Load PEAK | 6.2468 | 1.5276 | <0.0001* | 3.6637 | 2.0824 | 0.0840 |
| RVAs | O_2_ pulse PEAK | 0.3750 | 0.0815 | <0.0001* | 0.2244 | 0.1259 | 0.0800 |
| RVAs | RER PEAK | -0.0029 | 0.0017 | 0.0885 | 0.0015 | 0.0036 | 0.6733 |
| RVAs | VCO_2_ PEAK | 0.0619 | 0.0181 | 0.0008* | 0.0381 | 0.0245 | 0.1264 |
| RVAs | VE PEAK | 2.2198 | 0.7707 | 0.0046* | 1.9872 | 0.8283 | 0.0198 |
| RVAs | VO_2_ PEAK | 0.0598 | 0.0143 | <0.0001* | 0.0265 | 0.0212 | 0.2162 |
| RVAs | TV PEAK | 0.0246 | 0.0123 | 0.0481* | 0.0238 | 0.0168 | 0.1616 |
| RVAsI | BF PEAK | 1.1903 | 0.6227 | 0.0581 | 0.8461 | 0.6650 | 0.2085 |
| RVAsI | HR PEAK | -0.4400 | 0.6100 | 0.4720 | -1.0748 | 0.8052 | 0.1873 |
| RVAsI | Load PEAK | 10.8210 | 3.3630 | 0.0016* | 1.6781 | 3.4284 | 0.6264 |
| RVAsI | O_2_ pulse PEAK | 0.5227 | 0.1835 | 0.0051* | 0.0823 | 0.2075 | 0.6933 |
| RVAsI | RER PEAK | -0.0022 | 0.0037 | 0.5468 | 0.0039 | 0.0057 | 0.4959 |
| RVAsI | VCO_2_ PEAK | 0.0950 | 0.0399 | 0.0186* | 0.0101 | 0.0402 | 0.8033 |
| RVAsI | VE PEAK | 3.3611 | 1.6862 | 0.0483* | 1.7398 | 1.3775 | 0.2118 |
| RVAsI | VO_2_ PEAK | 0.0864 | 0.0320 | 0.0078* | -0.0021 | 0.0345 | 0.9527 |
| RVAsI | TV PEAK | 0.0042 | 0.0269 | 0.8770 | 0.0102 | 0.0273 | 0.7111 |
| RVD2 | BF PEAK | 0.1451 | 0.1905 | 0.4477 | 0.2942 | 0.2334 | 0.2128 |
| RVD2 | HR PEAK | -0.0397 | 0.1849 | 0.8302 | -0.5650 | 0.2769 | 0.0460* |
| RVD2 | Load PEAK | 1.8371 | 1.0440 | 0.0807 | 1.1256 | 1.1962 | 0.3508 |
| RVD2 | O_2_ pulse PEAK | 0.0964 | 0.0566 | 0.0904 | 0.1078 | 0.0715 | 0.1372 |
| RVD2 | RER PEAK | -0.0015 | 0.0011 | 0.1831 | -0.0015 | 0.0020 | 0.4582 |
| RVD2 | VCO_2_ PEAK | 0.0169 | 0.0122 | 0.1683 | 0.0112 | 0.0140 | 0.4272 |
| RVD2 | VE PEAK | 0.7764 | 0.5133 | 0.1327 | 0.8393 | 0.4772 | 0.0841 |
| RVD2 | VO_2_ PEAK | 0.0169 | 0.0098 | 0.0874 | 0.0103 | 0.0120 | 0.3951 |
| RVD2 | TV PEAK | 0.0108 | 0.0081 | 0.1848 | 0.0076 | 0.0096 | 0.4328 |
| RVIDd | BF PEAK | -0.0641 | 0.2146 | 0.7658 | 0.0023 | 0.2743 | 0.9933 |
| RVIDd | HR PEAK | -0.8613 | 0.1949 | <0.0001* | -0.7193 | 0.3184 | 0.0278* |
| RVIDd | Load PEAK | 5.2436 | 1.1010 | <0.0001* | 2.9657 | 1.3397 | 0.0309* |
| RVIDd | O_2_ pulse PEAK | 0.3471 | 0.0568 | <0.0001* | 0.2194 | 0.0792 | 0.0076* |
| RVIDd | RER PEAK | -0.0017 | 0.0012 | 0.1672 | -0.0057 | 0.0022 | 0.0120* |
| RVIDd | VCO_2_ PEAK | 0.0487 | 0.0132 | 0.0003* | 0.0267 | 0.0160 | 0.1000 |
| RVIDd | VE PEAK | 1.4425 | 0.5677 | 0.0122* | 0.2085 | 0.5674 | 0.7147 |
| RVIDd | VO_2_ PEAK | 0.0460 | 0.0104 | <0.0001* | 0.0288 | 0.0135 | 0.0370* |
| RVIDd | TV PEAK | 0.0318 | 0.0088 | 0.0004* | 0.0047 | 0.0111 | 0.6737 |
| RVWT | BF PEAK | -0.1247 | 0.8525 | 0.8839 | -1.0077 | 1.0882 | 0.3585 |
| RVWT | RER PEAK | -0.0042 | 0.0049 | 0.3990 | -0.0100 | 0.0091 | 0.2768 |
| RVWT | VCO_2_ PEAK | -0.0339 | 0.0550 | 0.5380 | 0.0716 | 0.0646 | 0.2728 |
| RVWT | VE PEAK | 1.6181 | 2.3076 | 0.4844 | 1.7023 | 2.2590 | 0.4543 |
| RVWT | TV PEAK | 0.0184 | 0.0363 | 0.6129 | 0.0696 | 0.0435 | 0.1155 |

Supplementary Table 3 shows the statistical analysis results of associations between cardiopulmonary exercise testing parameters and additional (not shown in the main table in the paper) structural parameters from echocardiography in sex groups. Values with asterisks represent *p*<0.05

Abbreviations: BF – breathing frequency; HR – heart rate; LAVI – end-systolic volume of left atrium indexed to body surface area (biplane measurement); LAVol – end-systolic volume of left atrium (biplane measurement); O_2_ pulse – the ratio of VO_2_ to HR; RAVI– end-systolic volume of right atrium indexed to body surface area (from 4-chamber view); RAVol – end-systolic volume of right atrium; RER – respiratory exchange ratio; RVAd – right ventricle area in diastole; RVAdI – right ventricle area in diastole indexed to body surface area; RVAs – right ventricle area in systole; RVAsI BSA – right ventricle area in systole indexed to body surface area; RVD2 – midcavity right ventricle dimension; RVIDd – right ventricle internal diameter in diastole; RVWT – right ventricle wall thickness; SD – standard deviation; TV – tidal volume; VCO_2­_ – the volume of produced CO_2_, VE – minute ventilation; VO_2_ – the volume of consumed O_2_;
